# Functional and behavioral effects of *de novo* mutations in calcium-related genes in patients with bipolar disorder

**DOI:** 10.1093/hmg/ddab152

**Published:** 2021-06-07

**Authors:** Takumi Nakamura, Kazuo Nakajima, Yuki Kobayashi, Shigeyoshi Itohara, Takaoki Kasahara, Takashi Tsuboi, Tadafumi Kato

**Affiliations:** Department of Psychiatry and Behavioral Science, Juntendo University Graduate School of Medicine, Tokyo 113-8421, Japan; Laboratory for Molecular Dynamics of Mental Disorders, RIKEN Center for Brain Science, Saitama 351-0198, Japan; Laboratory for Molecular Dynamics of Mental Disorders, RIKEN Center for Brain Science, Saitama 351-0198, Japan; Laboratory for Behavioral Genetics, RIKEN Center for Brain Science, Saitama 351-0198, Japan; Laboratory for Behavioral Genetics, RIKEN Center for Brain Science, Saitama 351-0198, Japan; Laboratory for Molecular Dynamics of Mental Disorders, RIKEN Center for Brain Science, Saitama 351-0198, Japan; Department of Life Sciences, Graduate School of Arts and Sciences, The University of Tokyo, Tokyo 153-8902, Japan; Department of Psychiatry and Behavioral Science, Juntendo University Graduate School of Medicine, Tokyo 113-8421, Japan; Laboratory for Molecular Dynamics of Mental Disorders, RIKEN Center for Brain Science, Saitama 351-0198, Japan

## Abstract

Bipolar disorder is a common mental illness occurring in approximately 1% of individuals and requires lifelong treatment. Although genetic factors are known to contribute to this disorder, the genetic architecture has not yet been completely clarified. Our initial trio-based exome sequencing study of bipolar disorder showed enrichment of *de novo*, loss-of-function (LOF) or protein-altering mutations in a combined group with bipolar I and schizoaffective disorders, and the identified *de novo* mutations were enriched in calcium-related genes. These findings suggested a role for *de novo* mutations in bipolar disorder. The validity of these statistical associations will be strengthened if the functional impact of the mutations on cellular function and behavior are identified. In this study, we focused on two *de novo* LOF mutations in calcium-related genes, *EHD1* and *MACF1*, found in patients with bipolar disorder. We first showed that the *EHD1* mutation resulted in a truncated protein with diminished effect on neurite outgrowth and inhibited endocytosis. Next, we used CRISPR/Cas9 to establish two knock-in mouse lines to model the *in vivo* effects of these mutations. We performed behavioral screening using IntelliCage and long-term wheel running analysis. *Ehd1* mutant mice showed higher activity in the light phase. *Macf1* mutant mice showed diminished attention and persistence to rewards. These behavioral alterations were similar to the phenotypes in previously proposed animal models of bipolar disorder. These findings endorse the possible role of *de novo* mutations as a component of the genetic architecture of bipolar disorder, which was suggested by the statistical evidence.

## Introduction

Bipolar disorder is a common mental disorder occurring in approximately 1% of individuals and is characterized by manic and depressive episodes (1–3) with lifetime prevalence. Genetic factors are known to be important in the pathogenesis of bipolar disorder (4). One genome-wide association study (GWAS) reported 30 loci significantly associated with bipolar disorder (5). Another recent GWAS reported 64 important loci (6). Recent sequencing studies have suggested possible roles for rare mutations, although the findings are equivocal (7), as is the role of copy number variation (8). Some rare mutations may cause somatic diseases that confer a risk of bipolar disorder (1,9–11). Though the role of polygenic architecture is well established, common variants can account for only 25% of the heritability (12). The contribution of other genetic factors to the genetic architecture of bipolar disorder is unknown.

**Figure 1:**
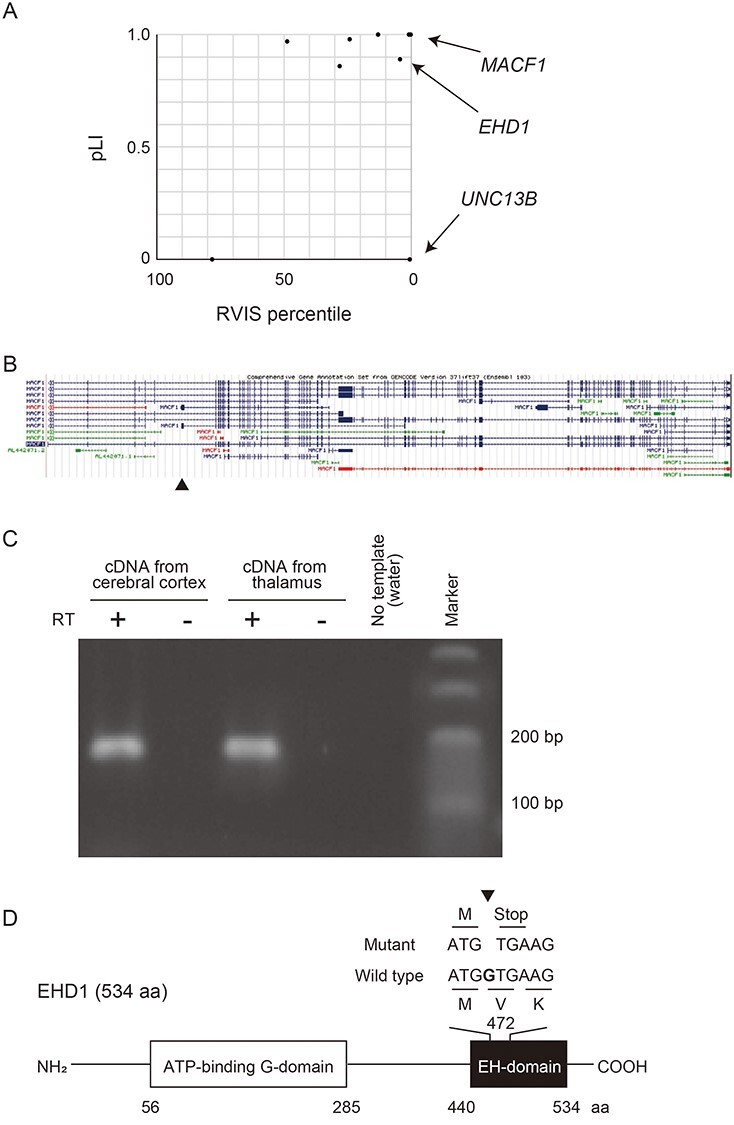
*De novo* LOF mutations reported in patients with bipolar disorder. (**A**) Plot of RVIS percentile and pLI of the genes in which *de novo* LOF mutations were previously found (13). Arrows show the genes coding calcium binding proteins (*MACF1*, *EHD1* and *UNC13B*). (**B**) Structures of *MACF1* isoforms. An arrowhead indicates the location of the *de novo* mutation on a minor exon. Data are retrieved from the UCSC database. (**C**) RT-PCR of the minor isoform of *MACF1*. The minor exon of *MACF1* in which the *de novo* mutation was found was amplified using cDNA derived from human cerebral cortex and thalamus. RT, Reverse-transcription. (**D**) Schematic diagram of the domain structure of EHD1 and the location of the *de novo* mutation of *EHD1*. The mutation, 1-bp deletion, was found in the last exon, and a truncated protein lacking most of the EH-domain is predicted to be expressed. aa, amino acids.

We previously reported the first study of *de novo* mutations in patients with bipolar disorder (13). In that report, we identified 71 ultra-rare *de novo* mutations in the probands, including nine loss-of-function (LOF) mutations. LOF and protein-altering mutations were significantly enriched in patients with bipolar I or schizoaffective disorder. *De novo* mutations in this population were enriched in genes encoding calcium ion binding proteins. This agreed with the enrichment of calcium channel genes from GWAS (6) and the association of bipolar disorder and psychoses with LOF mutations of *ATP2A2*, which encodes the endoplasmic reticulum Ca^2+^ pump (9). Among the three calcium-related genes affected by LOF mutations, *MACF1* and *EHD1* are of particular interest because they are intolerant to LOF and protein-altering mutations as evidenced by extreme residual variation intolerance scores (RVIS) (14) and probability of LOF mutation (pLI) (15) (Fig. 1A).

MACF1 is a member of the spectraplakin protein family and crosslinks microfilaments with microtubules (16,17). MACF1 regulates neural migration and vesicle transport (18,19). *Macf1*-null mice are early embryonic lethal, and *Macf1* brain-specific knockout mice exhibited multiple developmental defects of the brain such as disorganized cerebral cortex (20). *MACF1* is composed of 110 exons and has multiple alternative splicing isoforms. A recent study reported that several patients with brain malformation possessed *de novo* missense mutations or in-frame deletions of zinc-binding residues in the GAR domain of MACF1 (21). This also suggests that MACF1 is involved in the development of the nervous system. The mutation of *MACF1* found in the patient with bipolar disorder is a frameshift mutation (p.V266fs) in an exon specific for a splice variant (ENST00000496804), which is predicted to result in nonsense-mediated mRNA decay (NMD) (Fig. 1B and C).

EHD1 functions as a scaffold protein and binds to many proteins via an EH domain-containing EF-hand motif with calcium-binding properties (22). EHD1 regulates neurite outgrowth (23,24), vesicle transport (25,26), endocytosis and recycling of membrane proteins such as AMPA receptors (27). The neurite outgrowth contributed by EHD1 is important for the recovery after spinal cord injury (28). EHD1 is reported to play important roles in the development because *Ehd1* homozygous knockout mice show preweaning lethality (29). The *EHD1* mutation found in the patient with bipolar disorder is a frameshift mutation located in the last exon (Fig. 1D). This mutation causes a premature stop codon, and the mutant mRNA is predicted to escape NMD and generate a truncated EHD1 protein lacking the EH domain. Thus, the truncated protein may exert a dominant-negative effect.

The *de novo* mutations found in the patients with bipolar disorder were ultra-rare mutations which meant no other individual in the general population possessed the mutations, and it was difficult to predict the impacts of the mutations on the pathogenesis comparing with the healthy control by statistical analyses. We needed to analyze the functions of the mutations *in vitro* or *in vivo* experiments focusing on each mutation.

In this study, we first investigated the cellular functions, neurite outgrowth and endocytosis, of the truncated EHD1 protein caused by the *de novo* mutation. Second, we generated two heterozygous knock-in mouse lines with the mutations of *Macf1* and *Ehd1* using CRISPR/Cas9 and performed behavioral screening using IntelliCage (30) and long-term recording of wheel running (31,32), which has been used to assess bipolar disorder-like phenotypes of mouse models.

Here, we report that the mutation causing truncation of EHD1 results in LOF and dominant negative effects, and mutations in both EHD1 and MACF1 cause behavioral changes similar to those in previously proposed mouse models of bipolar disorder. These findings support the role of *de novo* mutations in the genetic architecture of bipolar disorder.

## Results

The *de novo* mutation investigated in *MACF1* (chr1: 39735167insC (hg19)) is a frameshift mutation located in an exon specific for one isoform (ENST00000496804) (Fig. 1B). Thus, we first determined whether or not the isoform was expressed in the human brain tissues using RT-PCR with the isoform-specific primer set. The analysis clearly demonstrated the expression of this isoform in the human cerebral cortex and thalamus (Fig. 1C).

**Figure 2:**
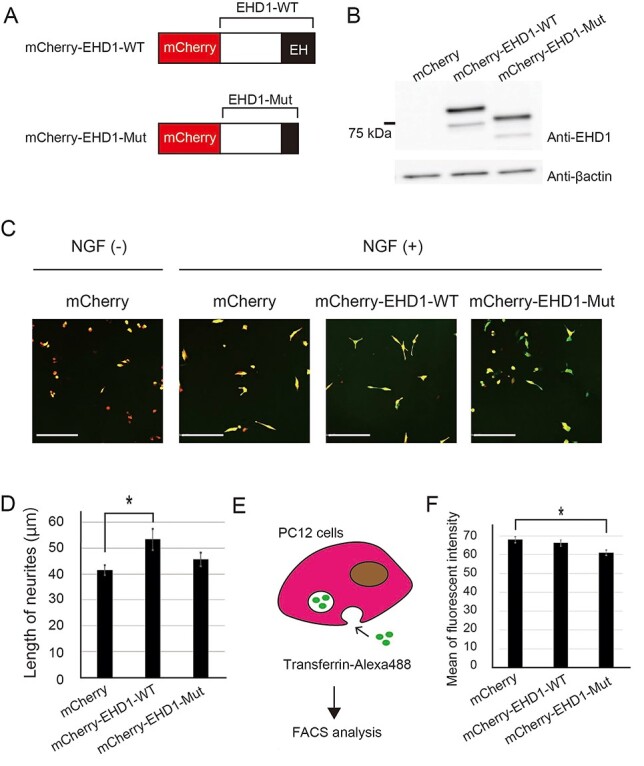
Functional analyses of the *de novo* LOF mutation in EHD1 in PC12 cells. (**A**) Diagram of the EHD1 proteins fused with mCherry. (**B**) Western blot for EHD1 in PC12 cells transfected with the EHD1 expression vectors. (**C**) Representative fluorescence microscope images of PC12 cells stimulated with β-NGF. mCherry-EHD1-(red) and Venus-(green) expression vectors were co-transfected into PC12 cells. The images of Venus were utilized for the analysis of the neurite length. Scale bars, 200 μm. (**D**) Neurite length. mCherry-EHD1-WT-expressing PC12 cells showed significantly longer neurites. *n* > 81 cells. Data shown are the mean ± standard error of the mean (SEM). Tukey’s multiple test was used for multiple comparisons. ^*^P < 0.05. Effect size, *η*^2^ = 0.027. (**E**) Schematic diagram of quantification of endocytosis. The uptake of Alexa Fluor 488-conjugated transferrin by starved PC12 cells was measured using FACS. (**F**) The mean of Alexa Fluor 488 fluorescent intensity measured by FACS. The fluorescent intensity was the average of Alexa Fluor 488 of mCherry positive cells. Transferrin uptake was significantly lower in mCherry-EHD1-Mut-expressing PC12 cells compared with mCherry-expressing cells. The trial was triplicated (*n* = 3). Data shown are the mean ± SEM. Tukey’s multiple test was used for multiple comparisons. ^*^P < 0.05. Effect size, *η*^2^ = 0.64.

The frameshift *de novo* mutation in *EHD1* (chr11: 64621996delC (hg19)) located in the last exon and is predicted to generate a truncated EHD1 protein lacking most of the EH domain (Fig. 1D). To investigate the function of truncated EHD1 at the cellular level, we constructed expression vectors for wild-type (WT) and mutant (Mut) EHD1 fused with mCherry (Fig. 2A and B) and overexpressed them in PC12 cells. We measured the neurite length with β-NGF stimulation (Fig. 2C). We found that PC12 cells expressing mCherry-EHD1-WT showed significantly longer neurites compared with control PC12 cells, while those expressing mCherry-EHD1-Mut did not show promoted neurite outgrowth (Fig. 2D).

Next, we investigated the effect of truncated EHD1 on vesicle transport, a process regulated by EHD1. PC12 cells transfected with mCherry-EHD1-WT- or -Mut-expression vectors were cultured in low-nutrient medium. We then exposed the starved PC12 cells to Alexa Fluor 488-labeled transferrin and quantified the fluorescence intensity of the cells by fluorescence-activated cell sorting (FACS) (Fig. 2E). We found that the uptake of transferrin by PC12 cells expressing mCherry-EHD1-Mut was lower than that of the control (Fig. 2F). This result suggested that mCherry-EHD1-Mut inhibited the endogenous endocytosis in PC12 cells in a dominant-negative manner.

**Figure 3:**
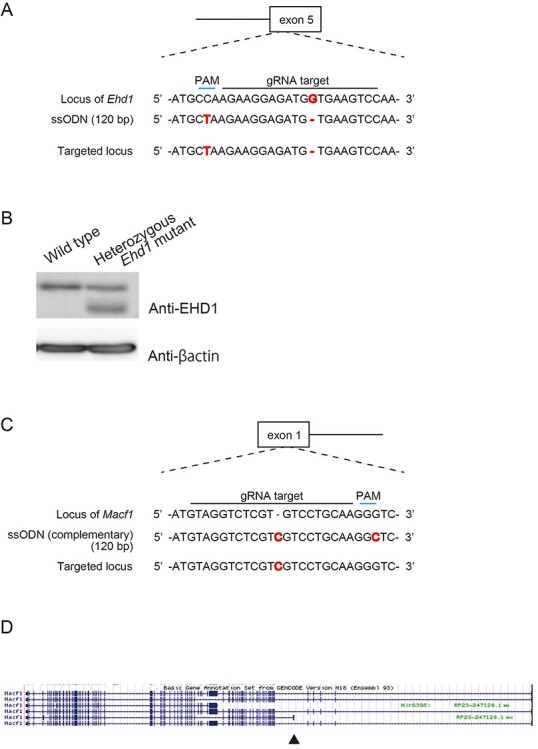
HDR-mediated genome editing to establish *Ehd1* and *Macf1* mutant mouse lines. (**A**) Sequences of *Ehd1* mutant mice. 1-bp deletion of G modeling the *de novo* mutation of the patient was introduced in the exon 5 of *Ehd1*. Sequence of ssODN utilized that of the strand in which PAM sequence exists. PAM, proto-spacer adjacent motif. (**B**) Western blot for EHD1 in the hippocampus of *Ehd1* mutant mice. The truncated protein was caused by the mutant allele of the heterozygous *Ehd1* mutant mice. (**C**) sequences of *Macf1* mutant mice. 1 bp insertion of C was introduced in the exon 1 of the minor isoform of *Macf1* to model the *de novo* mutation. (**D**) Splicing isoforms of *Macf1* in mice. The knock-in mutation on a minor exon of *Macf1* is indicated by an arrowhead.

To investigate whether these heterozygous mutations in *MACF1* and *EHD1* can cause behavioral changes, we generated knock-in mouse lines carrying each of these mutations (Fig. 3). We introduced 1 bp deletion into exon 5 of *Ehd1* and 1 bp insertion into exon 1 of *Macf1* modeling the *de novo* mutations via homology directed repair (HDR)-mediated genome editing using CRISPR/Cas9. We used whole exome sequencing of mutant F1 mice and verified no LOF mutations caused by off-target activity of Cas9. We then verified that truncated EHD1 protein was expressed in the hippocampus of heterozygous *Ehd1* mutant mice (Fig. 3B).

**Figure 4:**
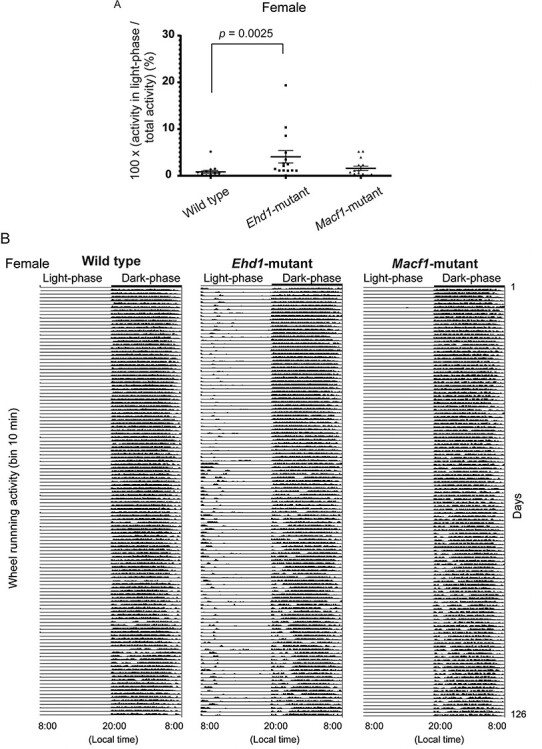
Higher activity in light-phase in a wheel running test of female mice. (**A**) *Ehd1* mutant female mice showed significantly higher rate of activity in light-phase per total activity. The test was performed for 18 weeks. Data shown are the mean ± standard error of the mean (SEM). The Kruskal–Wallis test was used for comparisons. *n* = 15 for each group. The *post-hoc* test was performed by Dunn’s multiple comparison test. WT versus *Ehd1* mutant; *P* = 0.0025. WT versus *Macf1* mutant; *P* = 0.40. Effect size, WT versus *Ehd1* mutant; *r* = 0.59, WT versus *Macf1* mutant; *r* = 0.23. (**B**) Representative actogram showing daily activity of each female mouse. The *Ehd1* mutant mouse exhibited high activities in light-phase. Each row means the data of one day. The *X*-axis indicates local time. *Y*-axis of each row shows the wheel running activity per 10 min.

We screened behavioral alterations in the knock-in mice by long-term wheel running analysis and the IntelliCage system, which can automatically perform multiple behavioral tests during group housing (Supplementary Material, Table S1). We did not find any manic- or depressive-like episodes in these two mouse lines in wheel running analysis (Supplementary Material, Fig. S1). However, *Ehd1* mutant female mice showed higher activity in the light phase than wild-type mice (Fig. 4). Male mice did not show a similar phenotype though we did not aim at analyzing male mice because we have noticed that the long-term wheel running activity recording of male C57/BL6 mice shows high variance and different patterns from female mice (Supplementary Material, Fig. S2).

**Figure 5:**
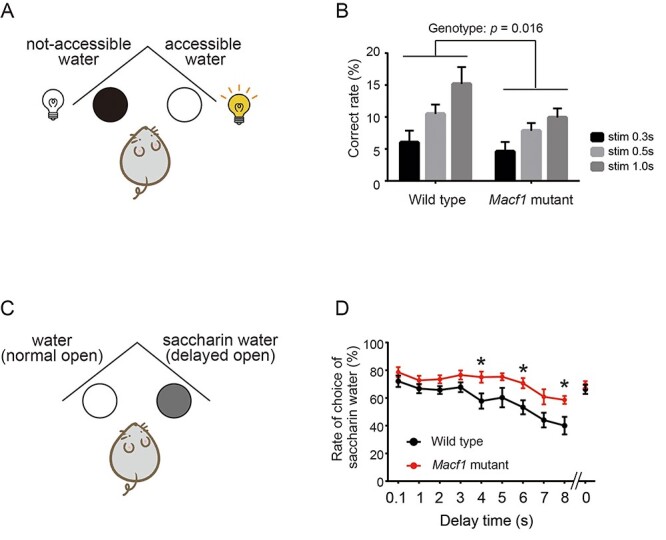
Significant behavioral alterations in *Macf1* mutant mice determined by IntelliCage analyses. (**A**) Schematic diagram of the attention test. Mice can drink water if they perform a nose poke in the gate on which an LED light was turned on. (**B**) Correct rate of male *Macf1* mutant mice in the attention test. *Macf1* mutant mice showed a significantly lower correct rate. Stim 0.3 s/0.5 s/1.0 s means the duration (0.3, 0.5 or 1.0 s) turning on the LED light on the door. Data shown are the mean ± standard error of the mean (SEM). Two-way analysis of variance (ANOVA) was used for multiple comparisons. *n* = 7 for each group. Interaction; *P* = 0.43, stimulation; *P* = 0.0001. Genotype; *P* = 0.016. Effect size, interaction; *η*^2^ = 0.025, stimulation; *η*^2^ = 0.095, genotype; *η*^2^ = 0.35. (**C**) Schematic diagram of the delay discounting test. The opening of the gate of saccharin water is delayed compared with the gate with water. The delay time between activation and opening increased by 1 s each day. (**D**) Rate of saccharin water selection by female *Macf1* mutant mice in the delay discounting test. Female *Macf1* mutant mice showed a significantly higher rate of choice of saccharin water even in long delay times. We measured the rate of choice of saccharin water at 0 s after all of the sessions to verify whether the preference of saccharin water remains or not. The data shown are the mean ± SEM. Two-way ANOVA was used for multiple comparisons. *n* = 7 for each group. Interaction; *P* = 0.14, Time; *P* = 0.0001, Genotype; *P* = 0.014. Effect size, Interaction; *η*^2^ = 0.035, Time; *η*^2^ = 0.30, Genotype; *η*^2^ = 0.16. The *post-hoc* test was performed by Bonferroni’s multiple comparison test. *P < 0.05.

Whereas the *Ehd1* mutant mice did not exhibit any significant changes in the IntelliCage behavioral tests, *Macf1* mutant mice showed characteristic behavioral changes in two tests of the IntelliCage analysis: attention and delay discounting (Fig. 5 and Supplementary Material, Figs S3–S6). Male *Macf1* mutant mice showed a lower correct rate in the attention test compared with wild-type mice (Fig. 5A and B). In the delay discounting test, the rate of choosing saccharin water in the sessions with longer wait time was higher in *Macf1* mutant female mice (Fig. 5C and D).

## Discussion

In this study, we focused on the *de novo* LOF mutations in Ca^2+^-related genes from patients with bipolar disorder which showed extreme pLI and RVIS scores (14,15). We found that these mutations caused alterations in cellular functions and behavior in mice. We validated that the frameshift mutation in *EHD1* resulted in a truncated EHD1 protein to be expressed. The mutant EHD1 lost the ability to promote neurite outgrowth and disrupted the endogenous endocytosis in PC12 cells. *Ehd1* mutant knock-in mice showed significantly higher activity in the light phase in wheel running analysis. We found that the isoform of *MACF1* disrupted by the patient’s mutation is expressed in the human brain and showed that *Macf1* knock-in mice carrying the mutation had reduced attention and persistence to rewards by IntelliCage analysis.

The higher activity in the light phase seen in the *Ehd1* mutant mice is similar to the phenotype of neuron-specific mutant *Polg* transgenic mice (31,32). *Polg* encodes a mitochondrial DNA polymerase, and the expression of mutant *Polg* resulted in the dysfunction of mitochondrial calcium signaling (33). The mutant *Polg* transgenic mice showed recurrent depression-like episodes and antidepressant-induced hyperactivity, and thus we proposed this line as a bipolar disorder mouse model.

On the other hand, *Macf1* mutant mice showed a lower correct rate in the attention test and a higher rate of choosing saccharin water in the delay discounting test. The results of the attention test suggest that *Macf1* mutant mice have a lower attention ability. Previous studies have revealed that cognitive impairment including poor performance in attention test is a part of clinical manifestations of bipolar disorder (34). The phenotype in the delay discounting test suggests either diminished delay discounting of reward or persistence to rewards. Diminished delay discounting of reward is a hallmark of depressive state (35) but the phenotype observed in the *Macf1* mutant mice is opposite to the phenomena observed in depressive patients. Impaired reward processing such as decreased reward-related brain activation has been reported in mania (36). Similar behavioral alteration in the delay discounting test has also been reported in *Ant1* knockout mice, where LOF mutations have also been identified in patients with bipolar disorder (37). The *Ant1* mutant mice also showed disrupted mitochondrial calcium signaling. Serotonin neuron-activated mice by optogenetics showed a similar phenotype (38), and *Ant1* mutant mice also displayed hyperactivity of serotonergic neurons. Although *Ant1* mutant mice did not show recurrent depression-like episodes, these findings suggested that loss of *Ant1* may be involved in a part of complex mechanisms of bipolar disorder, because serotonergic dysfunction has been implicated in bipolar disorder.

*Macf1* mutant mice showed sex differences in their behaviors (Fig. 5). Such behavioral differences depending on sex have been reported in mice and many mental disorders show sex differences in prevalence and/or symptomatology (39,40), and thus analyses in both sexes are recently recommended for preclinical research (41). Another possible reason why the mutant mice showed different patterns depending on the sex is the small sample size of IntelliCage analysis because this apparatus can analyze the limited number of mice at once. Indeed, the statistical power to detect the observed differences did not reach the general threshold, 0.8. Thus, a possibility of type I error cannot be excluded.

To date, the role of *de novo* mutations in bipolar disorder has been suggested mainly by statistical differences in the frequency of *de novo*, LOF or protein-altering mutations (13). This study is the first to reveal that these *de novo* mutations found in patients with bipolar disorder indeed cause behavioral alterations in mice. However, neither mutant mouse line captured manic- or depression-like episodes, and consistent behavioral alterations were not found in both mutant mouse lines. Bipolar disorder is not a simple genetic disease, but is considered a polygenic disorder with a complex genetic architecture (42). A combination of thousands of polymorphisms, rare mutations, copy number variations, *de novo* mutations, and possibly somatic mutations in the brain can cause bipolar disorder. Thus, the effect of a single genetic factor would be subtle, and we suppose that modeling a single genetic risk for bipolar disorder would be insufficient to induce perfect phenotypes of bipolar disorder and a single genetic risk such as a *de novo* mutation could cause only a part of phenotypes. Nevertheless, the behavioral phenotypes seen in these two mouse lines are seemingly capture one facet of myriad behavioral features of bipolar disorder.

Our study focusing on two calcium-related genes hit by *de novo* mutations support the well-established hypothesis that calcium signaling is important for the pathogenesis of bipolar disorder, supported by previous genetic (6,13) and animal model (32,37) studies. In conclusion, this is the first study to model the *de novo* mutations found in patients with bipolar disorder in mice and supports the role of *de novo* mutations in the genetic architecture of bipolar disorder.

## Materials and Methods

### Acquisition of pLI and RVIS

pLI results were acquired from the Exome Aggregation Consortium (ExAC) database. RVIS results were obtained from http://genic-intolerance.org.

### RT-PCR of an alternative *MACF1* exon

Human total RNA from the cerebral cortex and total mRNA from the thalamus were purchased from Takara Bio (Shiga, Japan; cerebral cortex, #636561; thalamus, #636135). RNA was reverse-transcribed using the SuperScript™ III First-Strand Synthesis System (Thermo Fisher Scientific, Waltham, MA, USA) with random hexamer primers. The specific exon of the minor isoform of *MACF1* was amplified using the following primer set with Ex Taq DNA polymerase (Takara Bio): forward primer, 5′-CACACATACATGGGATCCAGC-3′ and the reverse primer, 5′-CCACAGGAGTAGTTTCTCCTTGG-3′. PCR conditions were as follows: initial denaturation at 94°C for 1 min, followed by 35 cycles at 94°C for 30 s, 60°C for 30 s, and 72°C for 30 s, and a final extension step at 72°C for 5 min.

### Plasmid construction

We amplified DNA fragments of human EHD1-WT and -Mut using EHD1-WT and -Mut overexpression vectors (pcDNA3-Myc-EHD1 expression vector) previously reported as templates (13). The primer sets were as follows: forward primer, 5′-TTTTCTCGAGCTATGTTCAGCTGGGTCAGC-3′ and the reverse primer, 5′-TTTTGAATTCTCACTCATGTCTGCGCTTGG-3′. The fragments were inserted into the *Xho*I/*Eco*RI site of the pmCherry-C1 vector (Invitrogen, Carlsbad, CA, USA). The constructs were purified using PureLink™ HiPurePlasmid Filter Midiprep Kit (Invitrogen).

### Cell culture

PC12 cells were maintained in Dulbecco’s Modified Eagle Medium (D5796, Sigma-Aldrich, St. Louis, MO, USA; 11885, Gibco, Grand Island, NY, USA) containing the following reagents: 10% fetal bovine serum (FBS), 10% horse serum (Gibco), and 1× penicillin/streptomycin mixture (Nacalai Tesque, Inc. Kyoto, Japan) at 37°C in 5% CO_2_ condition.

### Transfection

48 h before transfection, PC12 cells were plated at 6 × 10^5^ cells per well in 6-well plates for western blotting, 5.0 × 10^5^ cells per well 35 mm glass bottom dishes (Matsunami, Ohsaka, Japan) for neurite outgrowth analysis, and 21 × 10^5^ cells on a 10 cm dish for endocytosis analysis. Next, 3.5 μg of the mCherry-EHD1 constructs (for western blot), 0.5 μg of the pVenus-C1 vector and 3.5 μg of mCherry-EHD1 constructs (for neurite outgrowth analysis), or 21.4 μg of the mCherry-EHD1 constructs (for endocytosis analysis) were transfected with 6.0 μl (for western blot and neurite outgrowth analysis) or 38 μl (for endocytosis analysis) of Lipofectamine 2000 (Invitrogen).

For protein extraction, transfected cells were lysed with 50 μl radioimmunoprecipitation (RIPA) buffer [0.15 M NaCl, 1% NP-40, 0.05% deoxycholate, 0.1% SDS, 0.05 M Tris–HCl (pH 8.0)] with complete Mini, EDTA-free (Roche, Upper Barvaria, Germany) at 4°C for 15 min. The lysate was centrifuged at 20 380 × *g* at 4°C for 15 min, and the supernatant was collected and saved. Dissected hippocampus samples were homogenized using a Dounce tissue grinder in 300 μl of lysis buffer [0.15 M NaCl, 1% NP-40, 1 mm deoxycholate, 10% glycerol, 50 mM Tris–HCl (pH 8.0) in sterile water] with complete Mini, EDTA-free (Roche). Protein concentrations were quantified using Micro BCA™ Protein Assay Kit (Thermo Fisher Scientific) according to the manufacturer’s instructions.

Next, 15 μg of protein from transfected cells, or 20 μg of protein from hippocampus samples, were separated using 8% SDS-polyacrylamide gel electrophoresis and transferred to PVDF membranes (Immoblion-P, Merck Millipore, Billerica, MA, USA). The membranes were then blocked with 5% skim milk in Tris-buffered saline (TBS) with 0.05% Tween-20 (TBST) for 30 min at 20–25°C, then incubated overnight at 4°C with rabbit anti-EHD1 (1:5000, ab109747; Abcam, Cambridge, UK) or mouse anti-β-actin (1:6000, A5441; Sigma-Aldrich) primary antibody in 5% skim milk. After washing five times with TBST for 5 min, the membranes were incubated for 1 h with horseradish peroxidase (HRP)-conjugated anti-rabbit-IgG for anti-EHD1 (1:5000, sc-2030; Santa Cruz Biotechnology, Dallas, TX, USA) or anti-mouse-IgG for anti-β-actin (1:6000, sc-2005; Santa Cruz Biotechnology) secondary antibody at 20–25°C. The immunoreactive bands were visualized using Amersham ECL Prime (GE Healthcare, Buckinghamshire, UK) and scanned using LAS-3000 image analyzer (Fujifilm, Tokyo, Japan).

### Measurement of neurite outgrowth

48 h after transfection, murine β-NGF (PEPROTECH Inc. (Rocky Hill, NJ, USA) was added to cell culture to a final concentration of 50 ng/ml. Green signals were imaged by a confocal microscopy (FLUOVIEW 1000; Olympus, Center Valley, PA, USA) 48 h after addition of β-NGF. We randomly selected 20 images per sample in each trial and trials were repeated in triplicate. We excluded neurites that were shorter than 13.4 μm in the statistical analyses because PC12 cells without β-NGF showed of the same length. Neurite length was measured using the Dendrite Autotrace mode in Neurolucida (MBF BIOSCIENCE, Williston, VT, USA). We analyzed mCherry-positive cells only.

### Analysis of endocytosis

After 48 h incubation post-transfection, cells were washed with 4 ml of 1× PBS after removal of the culture medium and incubated in starvation medium (0.1% BSA in DMEM) for 5 min in 5% CO_2_ at 37°C. After removing the starvation medium, cells were incubated with 1 ml of 0.25% trypsin–EDTA (Thermo Fisher Scientific) for 3 min at 37°C. Then, 9 ml of 1× PBS was added to the dish and the starved cells were collected. We obtained two 330 μl aliquots, which were named A and B.

Next, 330 μl of 8% PFA in PBS was added to aliquot A. After 10 min incubation at 20–25°C, the fixed cells were centrifuged for 3 min at 300 × *g* and suspended in 300 μl of 1× PBS. The cells in aliquot B were collected by centrifugation at 300 × *g* for 3 min. After removal of the supernatant, the cells were mixed with 300 μl of 50 μg/ml transferrin-Alexa Fluor 488 (Thermo Fisher Scientific) in DMEM. Transferrin-Alexa Fluor 488 uptake was induced by incubation for 20 min at 37°C in a water bath. The mixture was centrifuged for 3 min at 300 × *g* after incubation to collect the cells. The collected cells were fixed with 1 ml of 4% PFA/PBS for 10 min at 20–25°C. The fixed cells were suspended in 300 μl of 1× PBS in the same manner as aliquot A.

The fluorescence intensities of mCherry and Alexa Fluor 488 were measured with BD FACSAria (BD Bioscience, San Jose, CA, USA). The data were analyzed using FlowJo software (Digital Biology, Tokyo, Japan). The average fluorescence intensity of Alexa Fluor 488 was calculated using mCherry-positive cells whose threshold was defined by the data of un-stained cells.

### Design of guide RNA

Guide RNA (gRNA) for genome editing was designed using CRISPRdirect (http://crispr.dbcls.jp/) (Supplementary Material, Table S2A). The single guide RNA (sgRNA)-SpCas9-GFP all-in-one vector (pX458, Addgene) was generated, and the efficiency of cleaving the genome using the designed gRNA was evaluated by the Surveyor assay using the Surveyor Mutation Detection Kit (Integrated DNA Technologies, Coralville, IA, USA), as previously reported (29,43).

### Establishment of mutant mice

The sgRNA was first transcribed using MEGAshortscript Kit (Life Technologies, Carlsbad, CA, USA) and the sgRNA-SpCas9-GFP all-in-one vector as a template. A mixture of sgRNA (50 ng/μl), Cas9 mRNA (100 ng/μl, Sigma) and single strand oligo-deoxynucleotide (ssODN) (200 ng/μl, Supplementary Material, Table S2B) was microinjected into the cytoplasm of fertilized eggs obtained from C57BL6/N mice (CLEA Japan, Inc., Tokyo, Japan). The injected eggs were transferred into the oviducts of pseudopregnant ICR female mice.

### Genotyping of mutant mice

Each of *Macf1* and *Ehd1* mutant mouse line was maintained breeding with C57BL6/N (CLEA Japan, Inc.). The mice for behavioral tests were obtained by *in vitro* fertilization using sperms of the heterozygous mutant mice and eggs derived from C57BL6/N (CLEA Japan, Inc.). Genotyping of all mutant mice was performed using genomic DNA derived from tail tips. Tail biopsies were conducted on postnatal day 14, and each tail tip was incubated in 100 μl of lysis buffer (25 mm NaOH, 0.2 mm EDTA) at 95°C for 30 min. An equal volume of 40 mm TRIZMA hydrochloride was added to the lysate after incubation and the mixture was vortexed briefly. Afterward, 1 μl of the mixture was diluted with water 2.5-fold and were amplified by the PCR conditions shown in Supplementary Material, Table S2C. The PCR products were sequenced with BigDye Terminator V3.1 and ABI 3730xl sequencer (Life Technologies) using sequencing primers (Supplementary Material, Table S2D).

### Off-target analysis

Whole exome sequencing was performed to identify off-target mutations. Genomic DNA was purified from tail samples of F1 mutant mice using the GenElute Mammalian Miniprep kit (Merck Millipore). Whole exome sequencing and variant extraction were conducted by RIKEN Genesis. Off-target mutations were defined as those filtered by the following conditions as previously reported (44): (1) ‘known’ variant, (2) variant with <10 reads, (3) ‘missense’ variant, (4) variant found in the wild type, (5) homozygous variant, (6) variant with the ratio of reference sequence reads to alternative sequence reads <0.5 and (7) variant in a gene possessing more than three variants.

### Long-term recording of wheel-running activity

Wheel-running activity was recorded as previously described (32). We focused on the activities of female mice in this study because a previous study reported depressive-like episodes only in female mice. Fifteen mice per genotype were individually housed in cages (24 cm wide × 11 cm deep × 14 cm high) equipped with a running wheel (5 cm wide × 14 cm in diameter). Light–dark 12:12 h cycles (lights on at 08:00 local time) were controlled by a computer system (O’Hara & Co., Tokyo, Japan). Wheel-running activity was recorded using a PC system (O’Hara & Co.) for 18 weeks after habituation for more than 2 weeks. The light-phase activity (%) was defined as a percent of the activity during the light phase [12 h; 08:00–20:00 local time; zeitgeber time (ZT) 0–12] divided by the total activity during the dark phase (12 h; 20:00–08:00 the next day; ZT 12–24). The definition of depressive episodes was previously reported (32). Manic episodes were defined that the relative strength index (RSI) utilized in the previous report showed more than 75 at least for 1 day and more than 50 for consecutive 9 days.

### Behavioral test battery using IntelliCage

The IntelliCage system (TSE Systems, Inc. Chesterfield, MO, USA) was used as previously reported (45). IntelliCages (39 × 58 × 21 cm) contain four chambers in each corner accessible through an open doorway, which has a ring antenna and LED lights. Two doors in each chamber were controlled by computers and used to control access to water bottles. A radiofrequency identification transponder (Standard Microchip T-VA, DataMars, Lamone, Switzerland; and Trovan, Melton, UK) was implanted into the mouse dorso-cervical region under isoflurane inhalation anesthesia to track each mouse in the corner chambers. The light period was 08:00–20:00 local time, and the dark period was 20:00–08:00 (Light–Dark 12:12 h).

The schedule of the test battery is listed in Supplementary Material, Table S1. Seven wild-type and seven mutant mice of the same sex of each mouse line were kept and analyzed in each IntelliCage to avoid overcrowded breeding although at most 16 mice can be kept in one IntelliCage apparatus. The mice utilized in the IntelliCage analysis were independent individuals from those in the wheel running test. Testing began at 16 weeks of age. The details of each phase and test are as follows.

#### Free adaptation

All water bottles were always and freely accessible to allow mice to adapt to the IntelliCage.

#### Nose poke adaptation

Mice could drink water if they opened the gate that closed the bottles with a nose poke. The doors were open for 5 s after a nose poke. The gates could be opened anytime.

#### Drinking session adaptation

The gates were opened for 5 s at 21:00–24:00 local time after a nose poke.

#### Place learning test

Each mouse could drink in one of the four corners (correct corner) at 21:00–24:00 local time. The visit rates with nose pokes to the correct corner per corner were calculated every day.

#### Place learning reversal test

The correct corner was moved to the diagonally opposite corner in the place learning test. The correct rates were calculated in the same way as in the place learning test.

#### Impulsivity test

In the training step, doors were opened for 5 s if a nose poke was performed 1, 2 or 4 s after entering the chambers. In the test step, the doors were closed if the mice performed a nose poke within 2 s after entering into the chambers (premature nose poke). If a nose poke was performed for more than 2 s after entering, the doors opened. This test was accessible from 21:00 to 04:00 local time. Premature nose pokes were defined as ‘Incorrect’ nose pokes and omissions were defined as ‘Neutral’ nose pokes with a visit duration of less than 2 s. To calculate each rate, the number of each category was divided by the total number of nose pokes, which was the sum of the number of correct nose pokes, premature nose pokes and omissions.

#### Attention test

During the training step, one of the LED lights above the gates (correct) was turned on at random for 0.5, 1 or 2 s. After the nose poke to the ‘correct’ gate, the gate opened and the bottles were accessible. In the test step, one of the LED lights was turned on for 0.3, 0.5 or 1, randomly, 4 s after entering the chambers. The correct rate was calculated by the number of ‘correct’ nose pokes per the total number of nose pokes. The average of the correct rate over 5 days was utilized for statistical analyses.

#### Place avoidance test

On the first day, mice were exposed to an air puff if they visited one of the four corners (air puff corner). 24 h after the training, the mice were transferred to their home cages. Another 24 h after the transfer, all mice were returned to the IntelliCages. The visit rates to air puff corners per visits to corner were calculated as correct rates every day.

#### Delay discounting test

In this test, one of the two bottles in each corner contained 0.5% saccharin water, which is preferred by mice. The opening of the door to the bottle with saccharin water was delayed. In the training step, the delay time of saccharin water was 0 s to allow the mice to prefer saccharin water. In the test step, delays to open the doors were increased by 1 s every 24 h. The rate of drinking saccharin water was calculated daily. The bottles were accessible at all times.

## Supplementary Material

210520-supplementary_figure_1_ddab152

210520-supplemantary_figure_2_ddab152

210520-supplementary_figure_3_ddab152

210520-supplementary_figure_4_ddab152

210520-supplementary_figure_5_ddab152

210520-supplementary_figure_6_ddab152

Supplementary_tables_ddab152

supplementary_figure-legends_ddab152
