# Supplementary figures and images for: Functional and behavioral effects of de novo mutations in calcium-related genes in patients with bipolar disorder
Source: Hum Mol Genet. 2021 Jun 7;30(19):1851–62. doi: 10.1093/hmg/ddab152 (PMC8444452; doi:10.1093/hmg/ddab152)

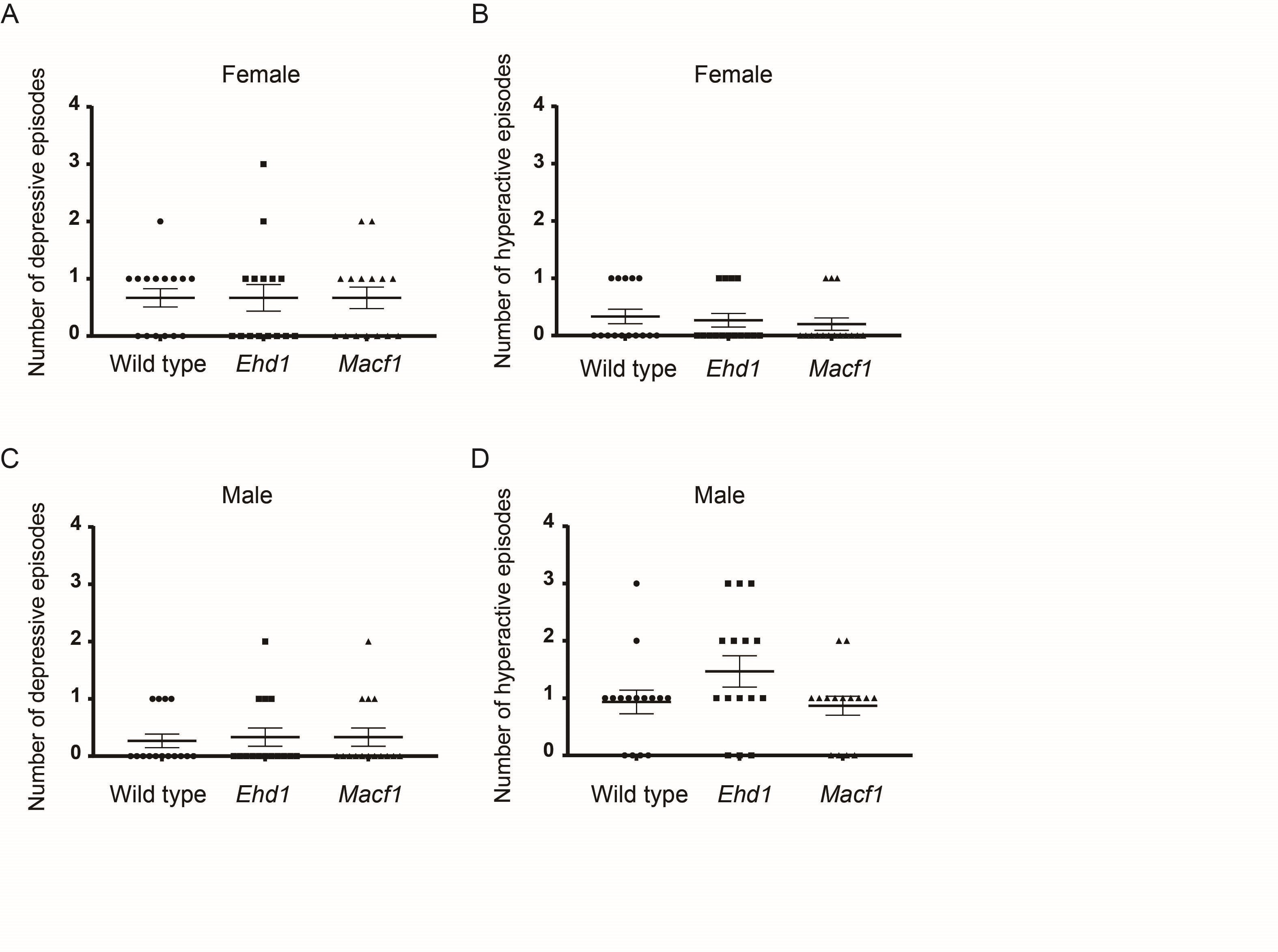

Supplement: 210520-supplementary_figure_1_ddab152 [file 210520-supplementary_figure_1_ddab152.jpeg]

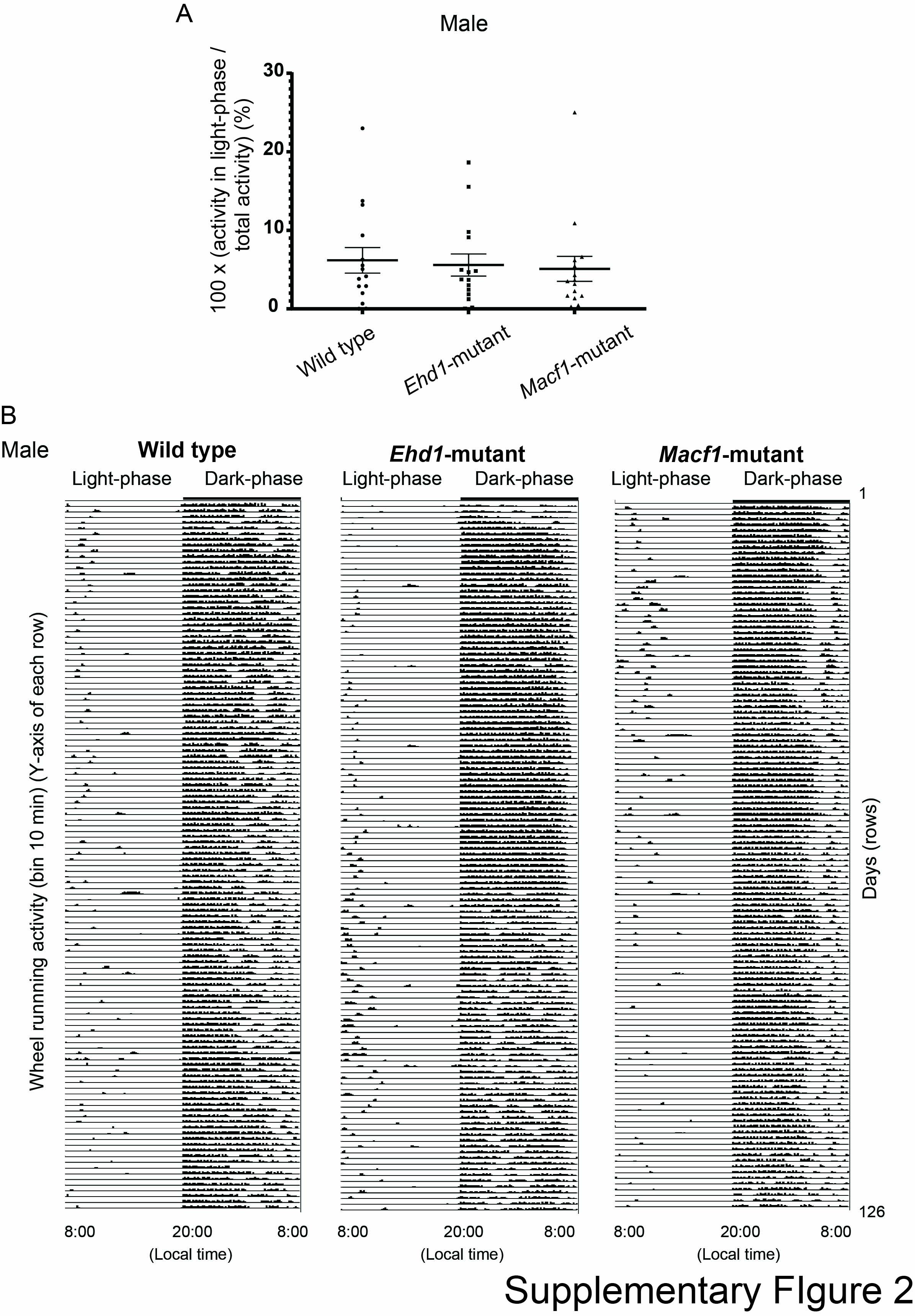

Supplement: 210520-supplemantary_figure_2_ddab152 [file 210520-supplemantary_figure_2_ddab152.zip › 210520-supplemantary figure 2.jpg]

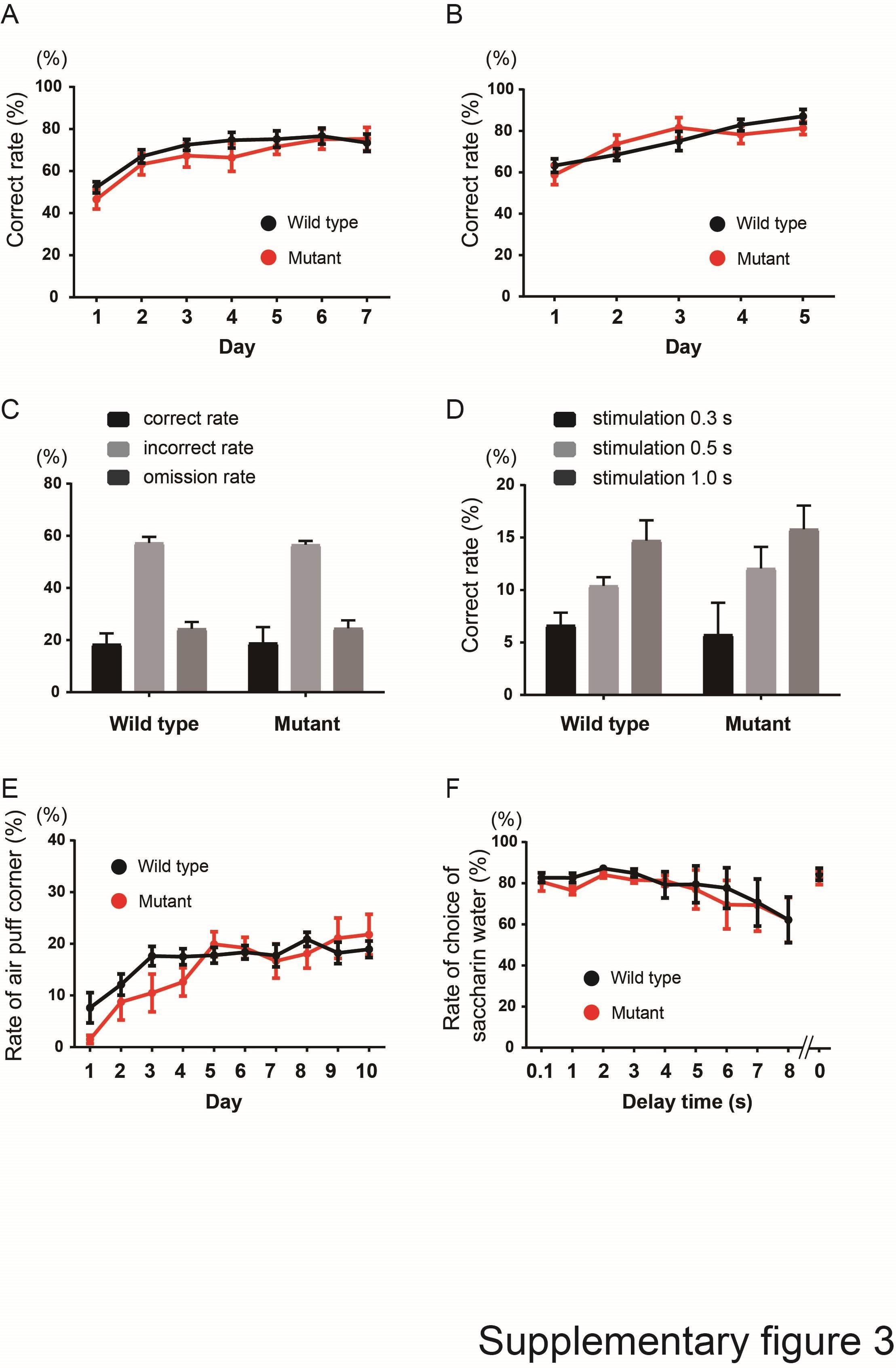

Supplement: 210520-supplementary_figure_3_ddab152 [file 210520-supplementary_figure_3_ddab152.jpeg]

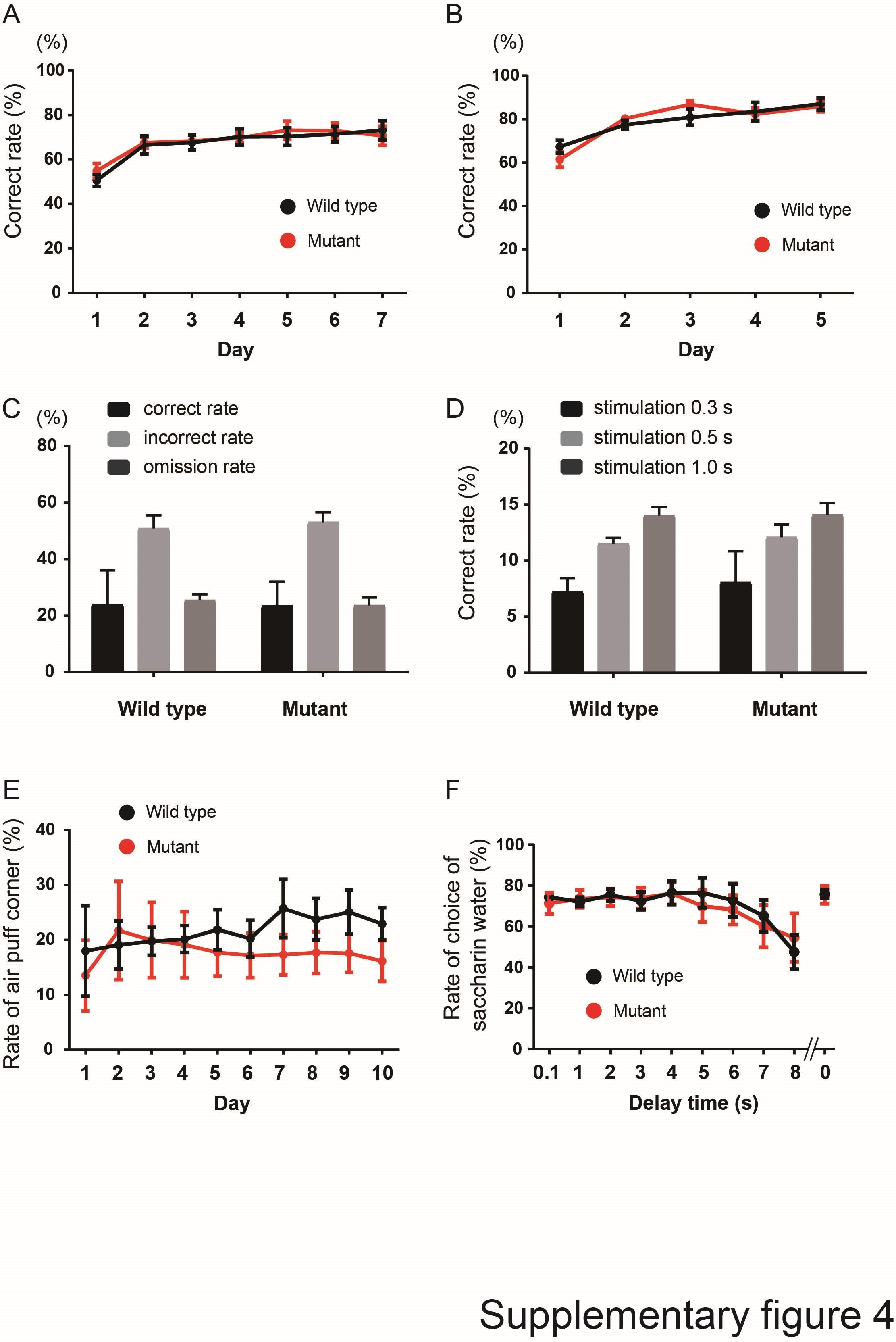

Supplement: 210520-supplementary_figure_4_ddab152 [file 210520-supplementary_figure_4_ddab152.jpeg]

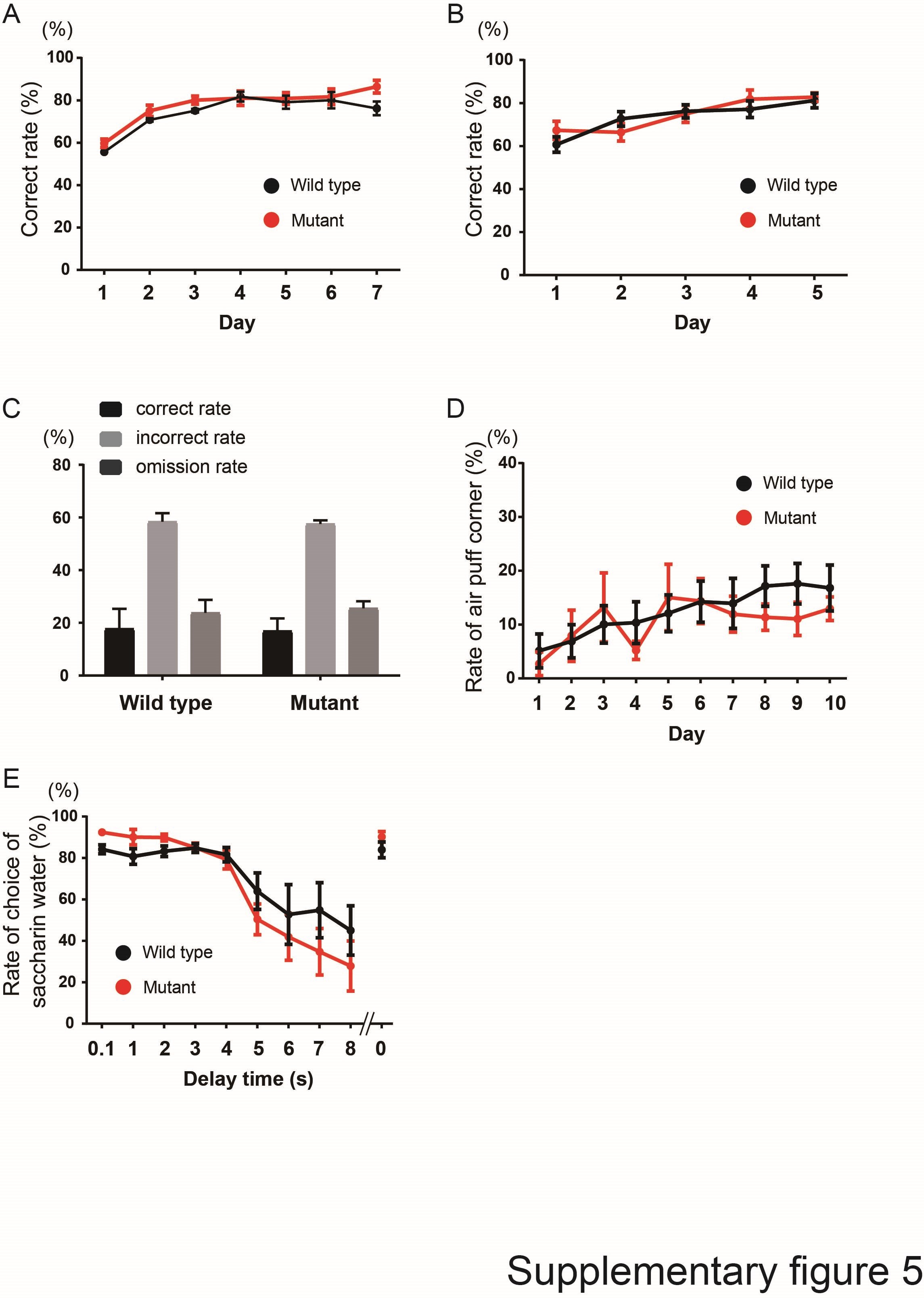

Supplement: 210520-supplementary_figure_5_ddab152 [file 210520-supplementary_figure_5_ddab152.jpeg]

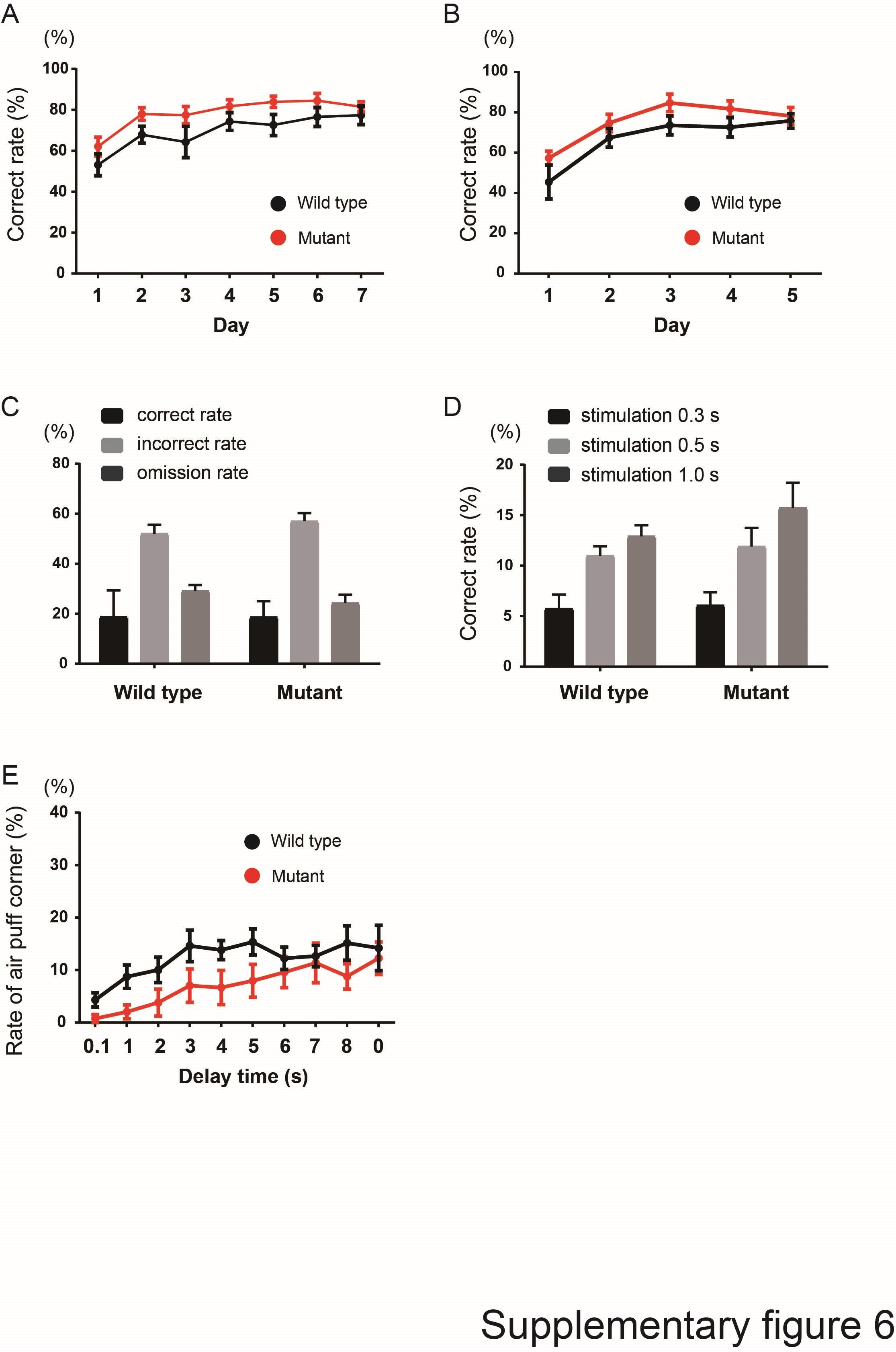

Supplement: 210520-supplementary_figure_6_ddab152 [file 210520-supplementary_figure_6_ddab152.jpeg]
