# Supplementary material for: Functional and behavioral effects of de novo mutations in calcium-related genes in patients with bipolar disorder
Source: Hum Mol Genet. 2021 Jun 7;30(19):1851–62. doi: 10.1093/hmg/ddab152 (PMC8444452; doi:10.1093/hmg/ddab152)
Supplement: Supplementary_tables_ddab152 [file supplementary_tables_ddab152.pdf]

Supplementary table 1. Schedule of IntelliCage analysis

Session name	Days
**Free adaptation**	2
**Nose poke adaptation**	3
**Drinking session adaptation**	3
**Place preference test**	7
**Place preference reversal test**	5
**Impulsivity test**	4
**Attention test**	5
**Place avoidance test**	9
**Delay discounting test**	10

Supplementary table 2. Primer sets in this study.

A. Primer sets for sgRNA (5'->3')

	top	bottom
Macf1	CACCGGTAGGTCTCGTGTCCTGCAA	AAACTTGCAGGACACGAGACCTACC
Ehd1	CACCGGGACTTCACCATCTCCTTCT	AAACAGAAGGAGATGGTGAAGTCC

B. ssODN for knock-in of mutations (5'->3')

Macf1	TGATGCCACTGACAGATAAATCACTCTGAGAAGCAGGCATTATGGGAGCCTTGCAGG ACGACGAGACCTACATGGGTAGGCTCTGTGTGAATAGGGTCTCCTGACTGTTTTGGTA TTGAT
Ehd1	TTCTACACACTGTCTCCTGTCAACGGCAAGATCACAGGTGCTAATGCTAAGAA GGAGATGTGAAGTCCAAGCTGCCCAACACAGTGCTGGGGAAGATCTGGAAGT TGGCAGATGTGGACA

C. Genotyping primers

	Fw	Rv	PCR conditions
Macf1	CAGGTAAAGGAGAGCTTCCAAGCAG	CACACTCCACTTCGATATGCCTGAG	94℃, 1 min → (98°C, 10 sec → 68°C , 1 min） ×30
Ehd1	CAGACCCAGGACTTCAGCAAGTTCC	GGATGAGTGTGGGGCTGTCAGACAG	

D. Sequencing primers for genotyping

	5'->3'
Macf1	CCTCTGAAACAGTAGATCAAGACCAACC
Ehd1	CCTCCGTTTGGAGGGTGGAATGAG
