# Supplementary material for: Functional and behavioral effects of de novo mutations in calcium-related genes in patients with bipolar disorder
Source: Hum Mol Genet. 2021 Jun 7;30(19):1851–62. doi: 10.1093/hmg/ddab152 (PMC8444452; doi:10.1093/hmg/ddab152)
Supplement: supplementary_figure-legends_ddab152 [file supplementary_figure-legends_ddab152.pdf]

Supplementary Figure 1. Episode measurements in wheel running test. For all panels, data shown are the mean ± standard error of the mean (SEM). The Kruskal-Wallis test was used for comparisons. n = 15 for each group. A, Number of depressive episodes of female mice in 18 weeks. $p = 0.92$. Effect size, WT vs *Ehd1* mutant; $r = 0.073$, WT vs *Macf1* mutant; $r = 0.024$. B, Number of hyperactive episodes of female mice in 18 weeks. $p = 0.72$. Effect size, WT vs. *Ehd1* mutant; $r = 0.075$, WT vs. *Macf1* mutant; $r = 0.15$. C, Number of depressive episodes of male mice in 18 weeks. $p = 0.99$. Effect size, WT vs. *Ehd1* mutant; $r = 0.020$, WT vs *Macf1* mutant; $r = 0.020$. D. Number of hyperactive episodes of male mice in 18 weeks. $p = 0.18$. Effect size, WT vs. *Ehd1* mutant; $r = 0.28$, WT vs. *Macf1* mutant; $r = 0.015$.

Supplementary Figure 2. Activity in light-phase in a wheel running test of male mice. A, There was no significance of the activities in light-phase. The test was performed for 18 weeks. Data shown are the mean ± standard error of the mean (SEM). The Kruskal-Wallis test was used for comparisons. n = 15 for each group. $p = 0.86$. Effect size, WT vs *Ehd1* mutant; $r = 0.038$, WT vs *Macf1* mutant; $r = 0.099$. B, Representative actogram showing daily activity of each male mouse. Each row means the data of one day. The X-axis indicates local time. Y-axis of each row shows the wheel running activity per 10 min.

Supplementary Figure 3. Results of IntelliCage of male *Ehd1* mutant mice. For all panels, data shown are the mean ± standard error of the mean (SEM). Two-way repeated measures analysis of variance (rmANOVA) was used for comparisons in A, B, E, and F. Two-way ANOVA was used for comparisons in C and D. A, Place learning test. n = 7 for each group. Interaction; $p = 0.81$. Time; $p < 0.0001$. Genotype; $p = 0.42$. Effect size, Interaction; $\eta^2 = 0.012$, Time; $\eta^2 = 0.37$, Genotype; $\eta^2 = 0.029$. B, Place learning reversal test. n = 7 for each group. Interaction; $p = 0.075$. Time; $p < 0.0001$. Genotype; p = 0.88. Effect size, Interaction; $\eta^2 = 0.042$, Time; $\eta^2 = 0.42$, Genotype; $\eta^2 =$

0.0011. C, Impulsivity test. n = 7 for each group. Interaction; $p = 0.94$. Pattern; $p < 0.0001$. Genotype; $p > 0.99$. Effect size, Interaction; $\eta^2 = 0.00021$, Pattern; $\eta^2 = 0.94$, Genotype; $\eta^2 = 7.53E-16$. D, Attention test. n = 7 for each group. Interaction; $p = 0.70$. Stimulation; $p < 0.0001$. Genotype; $p = 0.64$. Effect size, Interaction; $\eta^2 = 0.010$, Stimulation; $\eta^2 = 0.47$, Genotype; $\eta^2 = 0.0032$. E, Place avoidance test. n = 7 for each group. Interaction; $p = 0.16$. Time; $p < 0.0001$. Genotype; $p = 0.45$. Effect size, Interaction; $\eta^2 = 0.048$, Time; $\eta^2 = 0.36$, Genotype; $\eta^2 = 0.017$. F, Delay discounting test. WT; n = 6, Mut; n = 5. Interaction; $p > 0.99$. Time; $p = 0.0022$. Genotype; $p = 0.71$. Effect size, Interaction; $\eta^2 = 0.0064$, Time; $\eta^2 = 0.16$, Genotype; $\eta^2 = 0.0071$.

Supplementary Figure 4. Results of IntelliCage of female *Ehd1* mutant mice. For all panels, data shown are the mean ± standard error of the mean (SEM). Two-way repeated measures analysis of variance (rmANOVA) was used for comparisons in A, B, E, and F. Two-way ANOVA was used for comparisons in C and D. A, Place learning test. n = 7 for each group. Interaction; $p = 0.76$. Time; $p < 0.0001$. Genotype; $p = 0.80$. Effect size, Interaction; $\eta^2 = 0.0092$, Time; $\eta^2 = 0.36$, Genotype; $\eta^2 = 0.0038$. B, Place learning reversal test. n = 7 for each group. Interaction; $p = 0.11$. Time; $p < 0.0001$. Genotype; $p = 0.97$. Effect size, Interaction; $\eta^2 = 0.034$, Time; $\eta^2 = 0.55$, Genotype; $\eta^2 = 8.19E-5$. C, Impulsivity test. n = 7 for each group. Interaction; $p = 0.84$. Pattern; $p < 0.0001$. Genotype; $p > 0.99$. Effect size, Interaction; $\eta^2 = 0.0028$, Pattern; $\eta^2 = 0.71$, Genotype; $\eta^2 = 1.13E-15$. D, Attention test. n = 7 for each group. Interaction; $p = 0.90$. Stimulation; $p < 0.0001$. Genotype; $p = 0.49$. Effect size, Interaction; $\eta^2 = 0.0022$, Stimulation; $\eta^2 = 0.61$, Genotype; $\eta^2 = 0.0051$. E, Place avoidance test. n = 7 for each group. Interaction; $p = 0.84$. Time; $p = 0.90$. Genotype; $p = 0.47$. Effect size, Interaction; $\eta^2 = 0.019$, Time; $\eta^2 = 0.016$, Genotype; $\eta^2 = 0.025$. F, Delay discounting test. WT; n = 7, Mut; n = 5. Interaction; $p = 0.94$. Time; $p < 0.0001$. Genotype; $p = 0.87$. Effect size, Interaction; $\eta^2 = 0.014$, Time; $\eta^2 = 0.21$, Genotype; $\eta^2 = 0.0015$.

Supplementary Figure 5. Results of IntelliCage of male *Macf1* mutant mice. For all panels, data shown are the mean ± standard error of the mean (SEM). Two-way repeated measures analysis of variance (rmANOVA) was used for comparisons in A, B, D, and E. Two-way ANOVA was used for comparisons in C. A, Place learning testn = 7 for each group. Interaction; $p = 0.25$. Time; $p < 0.0001$. Genotype; $p = 0.17$. Effect size, Interaction; $\eta^2 = 0.023$, Time; $\eta^2 = 0.56$, Genotype; $\eta^2 = 0.031$. B, Place learning reversal test. n = 7 for each group. Interaction; $p = 0.29$. Time; $p < 0.0001$. Genotype; $p = 0.74$. Effect size, Interaction; $\eta^2 = 0.040$, Time; $\eta^2 = 0.34$, Genotype; $\eta^2 = 0.0023$. C, Impulsivity test. n = 7 for each group. Interaction; $p = 0.10$. Pattern; $p < 0.0001$. Genotype; $p > 0.99$. Effect size, Interaction; η2 = 0.0011, Pattern; η2 = 0.92, Genotype; η2 = 5.39E-16. D, Place avoidance test. n = 7 for each group. Interaction; $p = 0.50$. Time; $p < 0.0001$. Genotype; $p = 0.67$. Effect size, Interaction; $\eta^2 = 0.027$, Time; $\eta^2 = 0.12$, Genotype; $\eta^2 = 0.0080$. E, Delay discounting test. n = 7 for each group. Interaction; $p = 0.061$. Time; $p = 0.0001$. Genotype; $p = 0.66$. Effect size, Interaction; $\eta^2 = 0.038$, Time; $\eta^2 = 0.53$, Genotype; $\eta^2 = 0.0038$.

Supplementary Figure 6. Results of IntelliCage of female *Macf1* mutant mice. For all panels, data shown are the mean ± standard error of the mean (SEM). Two-way repeated measures analysis of variance (rmANOVA) was used for comparisons in A, B, and E. Two-way ANOVA was used for comparisons in C and D. A, Place learning test. n = 7 for each group. Interaction; $p = 0.88$. Time; $p < 0.0001$. Genotype; $p = 0.074$. Effect size, Interaction; $\eta^2 = 0.0092$, Time; $\eta^2 = 0.28$, Genotype; $\eta^2 = 0.10$. B, Place learning reversal test. n = 7 for each group. Interaction; $p = 0.80$. Time; $p < 0.0001$. Genotype; $p = 0.078$. Effect size, Interaction; $\eta^2 = 0.011$, Time; $\eta^2 = 0.40$, Genotype; $\eta^2 = 0.066$. C, Impulsivity test. n = 7 for each group. Interaction; $p = 0.23$. Pattern; $p < 0.0001$. Genotype; $p > 0.99$. Effect size, Interaction; $\eta^2 = 0.014$,

Pattern; $\eta^2 = 0.82$, Genotype; $\eta^2 = 3.16E-16$. D, Attention test. n = 7 for each group. Interaction; $p = 0.66$. Stimulation; $p < 0.0001$. Genotype; $p = 0.25$. Effect size, Interaction; $\eta^2 = 0.011$, Stimulation; $\eta^2 = 0.49$, Genotype; $\eta^2 = 0.018$. E, Place avoidance test. n = 7 for each group. Interaction; $p = 0.44$. Time; $p < 0.0001$. Genotype; $p = 0.12$. Effect size, Interaction; $\eta^2 = 0.021$, Time; $\eta^2 = 0.17$, Genotype; $\eta^2 = 0.10$.
